# Supplementary material for: The Effect of Microwave Radiation on the Green Color Loss of Green Tea Powder
Source: Foods. 2022 Aug 22;11(16):2540. doi: 10.3390/foods11162540 (PMC9407078; doi:10.3390/foods11162540)
Supplement: Supplementary file 1 [file foods-11-02540-s001.zip › foods-1835463-supplementary.pdf]

**Supplementary Materials**

# The effect of microwave radiation on the green color loss of green tea powder

Huijuan Wang†, Yan Zhu†, Dongchao Xie, Haihua Zhang, Yahui Zhang, Peng Jin*, Qizhen Du*

*Department of Food Science and Technology, The College of Food and Health, Zhejiang A & F University, Linan, 311300, China*

† These authors contributed equally.

* Corresponding Author: The College of Agricultural and Food Sciences, Zhejiang A & F University, Hangzhou 311300, China. Tel.: +86-571-63742176; Fax: +86-571-88218710. E-mail addresses: jinpeng@zafu.edu.cn (P. Jin), qizhendu@163.com (Q. Du).

*1 Color spectrum*

The color spectrum in CIE system in the second phase limit include yellow and green (Figure S1).

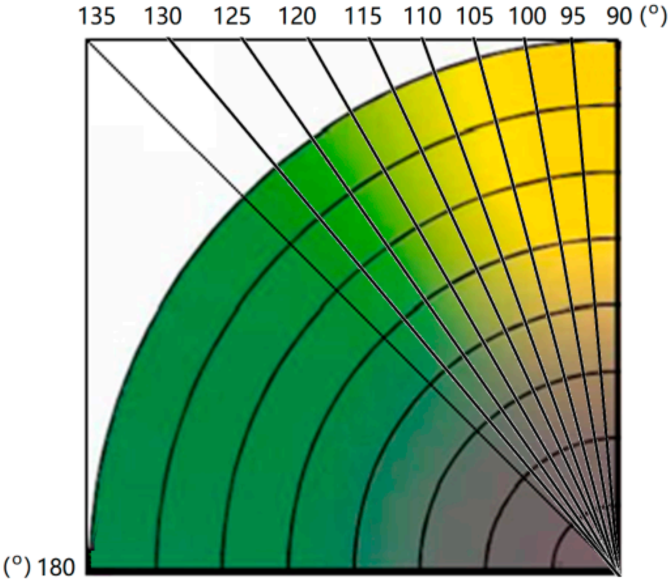


**Figure S1** The color spectrum corresponding Hue angle 90–135°

*2 Calibration of L, a, and b values*

A spectrophotometer is usually used to measure L, a, and b values for various samples. However, it could not provide actual values for powdery samples because light scatters among powdery particles. Therefore, the measured L, a, and b values of green tea powder must be calibrated.

After color pictures of green tea powder treated with a series of microwave radiation were obtained, the color was matched with the SLcolor software, which could give the corresponding L, a, and b values (Figure S2). The average values of the ratio of

matched value/measured value for L, a, and b for six green tea powder samples were 0.951 ± 0.080, 2.293 ± 0.017, and 1.115 ± 0.075 (n = 6), respectively, indicating that the calibrated values (L*, a*, and b*) from the measured L, a, and b values could be obtained by the calibration coefficients 0.951, 2.293, and 1.115, respectively.

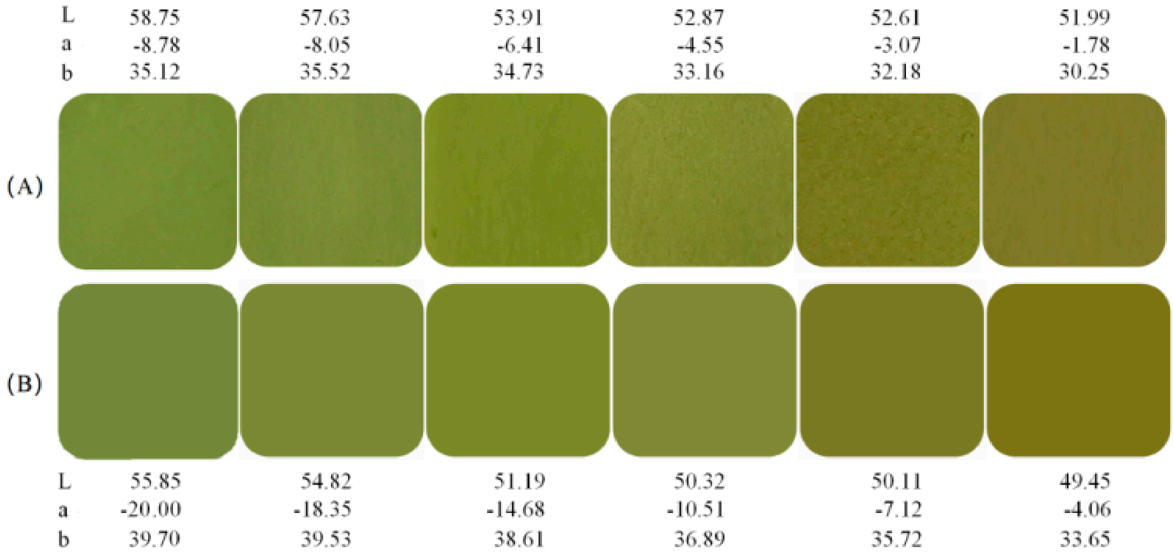


**Figure S2** The color and measured L, a, and b value (A) and the corresponding matched color and values of L, a and b given by software for the GTP samples (B).
